# Effects of fitspiration on believability and intention to exercise

**DOI:** 10.3389/fpsyg.2023.1094276

**Published:** 2023-02-20

**Authors:** Tanya R. Berry, Sean Locke, Elaine M. Ori

**Affiliations:** ^1^Faculty of Kinesiology, Sport, and Recreation, University of Alberta, Edmonton, AB, Canada; ^2^Department of Kinesiology, Faculty of Applied Health Sciences, Brock University, St. Catharines, ON, Canada

**Keywords:** fitspiration, implicit attitude, believability, explicit attitude, cognitive errors, intention

## Abstract

**Introduction:**

Although Fitspiration is purportedly intended to motivate people to be fit and healthy, a body of research has demonstrated negative effects of these media in men and women. Understanding mechanisms can help create more targeted interventions aimed at mitigating the negative effects of Fitspiration. This research examined if selected implicitly or explicitly measured constructs moderated or mediated the effects of Fitspiration. The purposes were to examine the believability (finding the media truthful) of Fitspiration (study one; data from 139 women and 125 men aged 18–33 years were analyzed), the effects of Fitspiration on exercise intention (study two; data from 195 women and 173 men aged 18–30 years were analyzed), and whether these effects were moderated by exercise-related cognitive errors (negatively biased perception of exercise) or mediated by implicit (evaluative responses to stimuli) or explicit (reasoned evaluation of stimuli) attitudes.

**Methods:**

In two separate studies, self-identified men and women first completed a measure of exercise-related cognitive errors, then viewed gender-specific Fitspiration media, followed by measures of implicit attitudes, explicit attitudes, believability, and demographics. In study two, participants were randomly assigned to Fitspiration or control media conditions and also completed measures of fitspiration-related cognitive errors and intention to exercise. In the first study, one model was tested for each gender sample. It was hypothesized that implicit and explicit attitudes would be positively related to believability, and that exercise-related cognitive errors would moderate these relationships. In study two, separate models were tested with exercise-related or Fitspiration-related cognitive errors as the moderators with each gender sample. It was hypothesized that implicit attitudes, explicit attitudes, and believability would be positively related to intention, that the control media would lead to greater intention to exercise than the Fitspiration media, and that exercise-related cognitive errors and Fitspiration-related cognitive errors would moderate these relationships.

**Results:**

The majority of hypothesized relationships were not supported. A negative relationship between exercise-related cognitive errors and believability was found.

**Discussion:**

Overall, these studies identify and exclude factors that predict Fitspiration believability and the role that factors such as cognitive errors and attitudes may play in that.

## 1. Introduction

Maibach (2007) identified a complex and contradictory exercise-related media landscape, which has arguably become even more complicated since then. The popularity of social media has changed media consumption and given rise to trends such as Fitspiration websites and hashtags (e.g., #fitspiration, #fitspo) on platforms such as Instagram. Content analyses demonstrate that Fitspiration shows idealized bodies as desirable and achievable through exercise along with text that is often about appearance (Boepple et al., 2016; Tiggemann and Zaccardo, 2018). The women’s bodies on display are thin and toned and the men’s bodies are very muscular with little body fat. The sexual objectification of women’s bodies is also common, as is stigmatizing overweight and obesity, and supporting disordered eating. There is often accompanying text such as “be stronger than your excuses,” which reinforce ideas that to achieve such bodies requires sacrifice and hard work.

Although Fitspiration is purportedly intended to motivate people to be fit and healthy (Boepple et al., 2016), a body of research has demonstrated negative effects of these media in men and women. Griffiths and Stefanovski (2019) reported that viewing Fitspiration was related to lower body satisfaction and lower positive affect in men and women. Men who view more Fitspiration have higher tendencies to internalize muscular ideals and to compare their appearance to others; the latter was related to less motivation to exercise for health (Fatt et al., 2019). Men have also reported lower mood and increased desire for more muscles after seeing Fitspiration (Yee et al., 2020). Women exposed to Fitspiration rated themselves as less attractive and had lower weight satisfaction than those viewing control images of travel destinations (Fioravanti et al., 2021). Exposure to Fitspiration may also be related to higher inspiration to be fit, but not actual immediate exercise behavior (Prichard et al., 2020). Another study found that Fitspiration promoting an athletic ideal (i.e., both thin and fit) contributed to greater body dissatisfaction in women than those featuring thin or muscular ideals (Robinson et al., 2017).

Despite this body of work, the mechanisms through which Fitspiration may influence consumers are still not completely understood (Vandenbosch et al., 2022). Understanding mechanisms can help create more targeted interventions aimed at mitigating the negative effects of Fitspiration. When conducting such research, it is necessary to examine if there are differences in effects between men and women exposed to gender-specific Fitspiration. Wood and Eagly’s (2012) biosocial constructionist theory recognizes an interaction between biology and psychology such that ability to psychologically adapt is influenced by constraints defined by sex (e.g., reproductive capabilities or typically larger musculature and strength in men) that varies across culture. This is reinforced through societal stereotypes and expectations such that many adults internalize beliefs about what women or men “should” be like, resulting in behaviors more likely to conform to cultural expectations (Eagly and Wood, 2013). There is meta-analytic evidence that media depicting gender-normed bodies have negative effects for women and men, likely due to how men and women are depicted in the media and subsequent socialization (Scharrer, 2013). At the same time, a meta-analysis has shown that men have higher body appreciation than women although the result was greater for college compared to community adult samples and effect sizes were small (He et al., 2020). Therefore, the research reported here examined if selected constructs, measured either implicitly or explicitly, may moderate or mediate the effects of Fitspiration across two studies with self-identified women and men. A main construct measured was the believability of Fitspiration media because although one study found that factually incorrect blog posts targeting appearance in women were rated as more believable (Ori et al., 2021), there is no known research specifically examining the believability of Fitspiration media in men or women. Therefore, the first study focused on how believable people find Fitspiration, and the second study replicated and extended the first study to examine the effects of Fitspiration on intention to exercise. Specific mediators and moderators of the believability of these media and the effects of these media on intention to exercise were selected for examination as outlined below.

**Figure 1:**
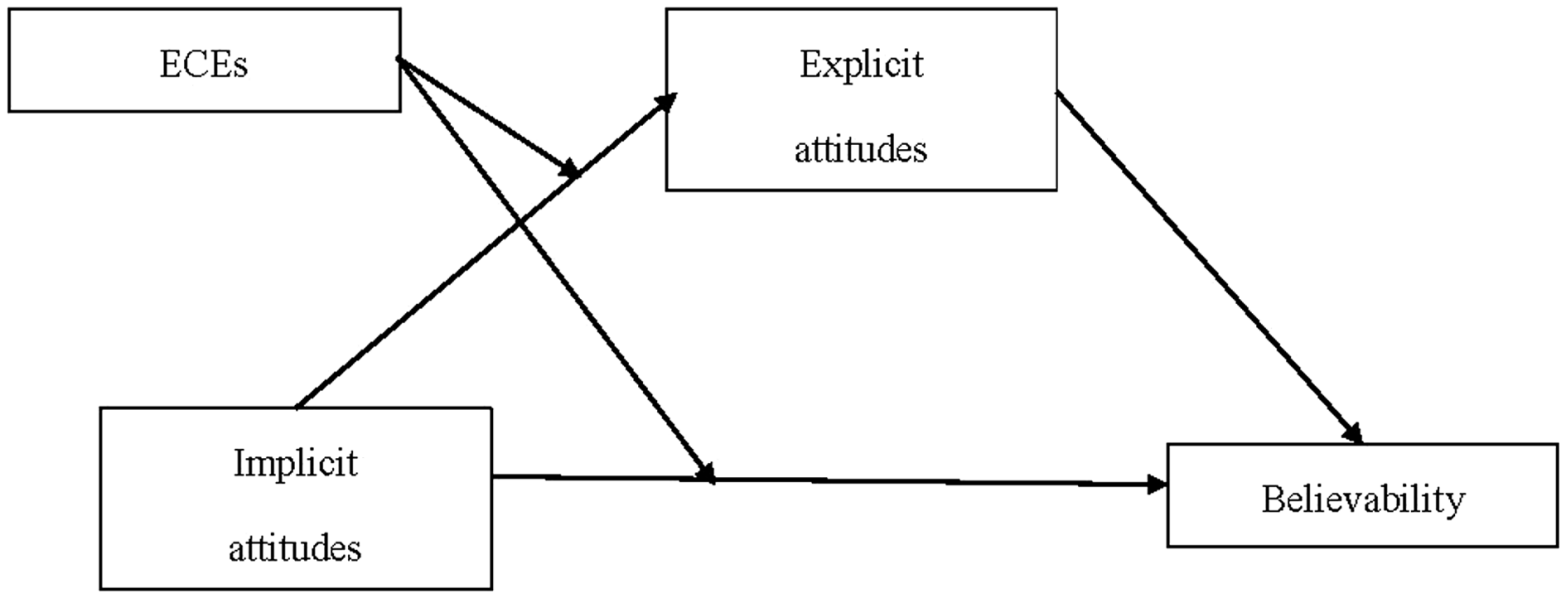
Model tested in study one.

**Figure 2:**
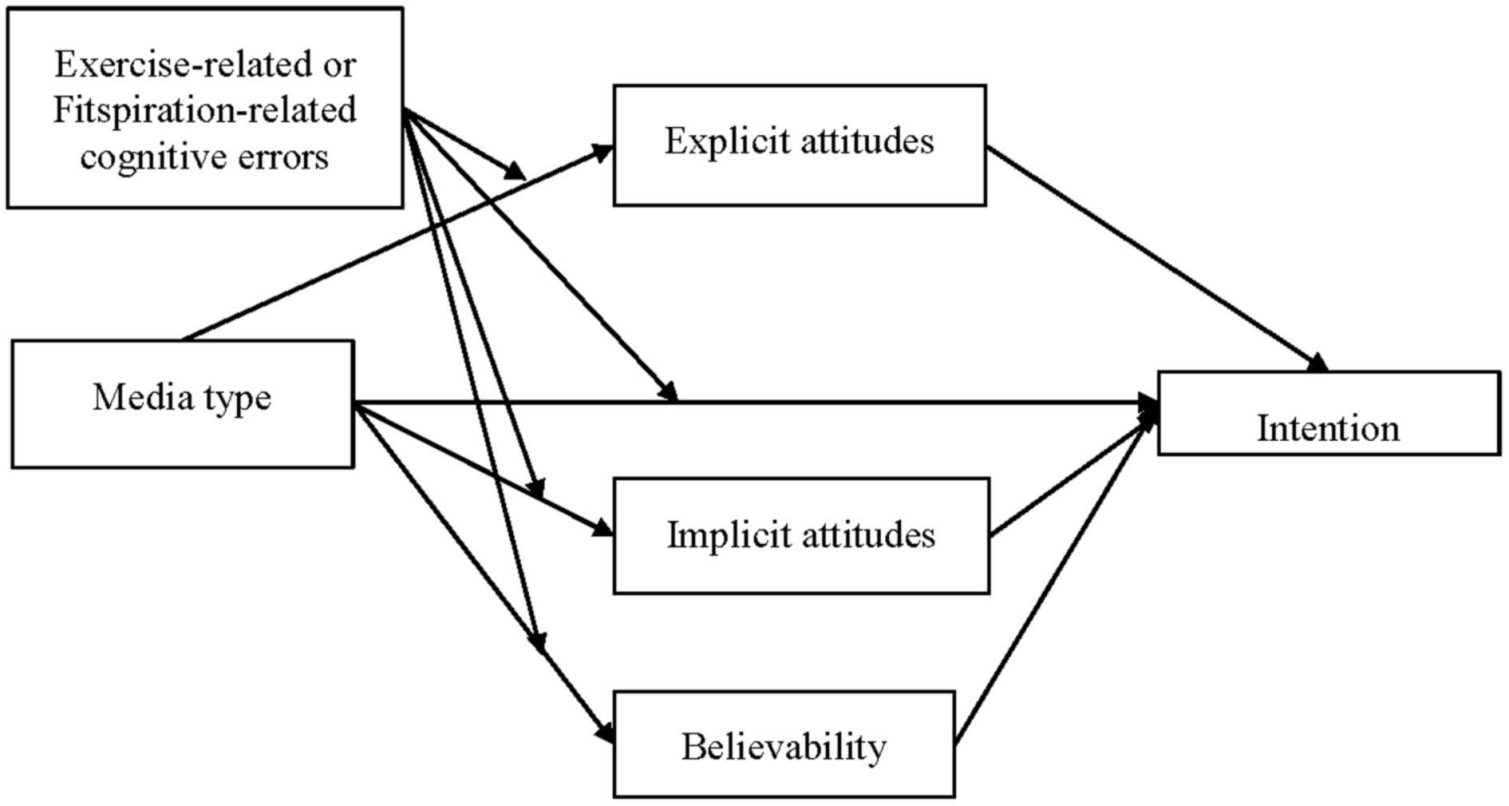
Model tested in study two.

Because Fitspiration media are extreme in types of bodies shown and the language used, they may elicit both implicitly (i.e., impulsive) and explicitly (i.e., reflective) measured attitudinal responses. It is also possible that people who have distorted views of exercise (i.e., make exercise-related cognitive errors) may view Fitspiration differently or find it more believable. Therefore, the first study investigated impulsive evaluations of exercise (operationalized as implicit attitudes) and reflective processes including cognitive errors and explicit attitudes after viewing Fitspiration and their relationship to believability. As shown in Figure 1, and further described in the following sections, exercise-related cognitive errors were measured as a possible moderator of explicit attitudes and believability and the relationship between implicit attitudes and believability. Study 2 was an experimental study that tested Fitspiration media compared to control media and examined the effects of these on intention to exercise with implicit attitudes, explicit attitudes, and believability examined as possible mediators, and cognitive errors as a possible moderator (see Figure 2).

Believability refers to whether people find the actual content of messages to be truthful and trustworthy (Beltramini and Evans, 1985). Research by O’Cass and Griffin (2006), who used Beltramini and Evan’s believability measure, found that the more believable participants rated anti-smoking or anti-excessive drinking advertisements to be, the stronger participant’s attitudes were in line with the advertisement’s messages (e.g., they reported disliking smoking). These authors also reported that the believability of the advertisements was related to intention to comply with the message. Others have found that a factually incorrect blogpost about the benefits of resistance training for appearance in women was rated as more believable than the same blog post corrected to only include evidence-based information (Ori et al., 2021). The incorrect blogpost included some information that may be seen in women’s Fitspiration such as the need to restrict calories, but the effects of Fitspiration on believability have yet to be examined.

Implicit attitudes are evaluative responses to stimuli that arise very quickly and more automatically. They are measured using tasks that constrain responses, for example by giving people very little time to respond (Gawronski and De Houwer, 2014). Implicit attitudes may be created after repeatedly seeing bodies such as those shown in Fitspiration paired with the concept of exercise (i.e., a strong association develops between certain body types and exercise, regardless of how truthful the association is). Implicit attitudes may also reflect automatically activated propositions that are considered truthful (e.g., exercise is something I want to do; Deutsch et al., 2017). Very little research has examined implicit attitudes after viewing Fitspiration but such media may elicit implicit exercise-related attitudes. For example, researchers have found that participants who viewed e-cigarette advertising had higher implicit attitudes toward e-cigarettes compared to participants who viewed control advertisements (Pokhrel et al., 2019).

Explicit attitudes can be conceptualized as how something is evaluated, be it favorably or unfavorably (De Houwer et al., 2013). Given that many young adults have been previously exposed to Fitspiration (Griffiths and Stefanovski, 2019), they likely already hold some form of attitude about it. Because O’Cass and Griffin (2006) found that believability of advertisements was related to attitudes and subsequent intention to comply with advertising messages, the effects of explicit attitudes toward Fitspiration were measured in the current research. Moreover, researchers have found that implicitly measured believability of exercise messages was related to attitudes and believability (Berry et al., 2011) and thus explicit and implicit attitudes are important constructs to measure in relation to each other.

Deutsch et al. (2017) describe how impulsive, often emotional responses may underpin cognitive issues such as being overly reflective (e.g., through rumination or over-thinking issues). The cognitive errors framework addresses some possible consequences of being overly reflective wherein cognitive errors occur because individuals perceive situations through a distorted or biased lens (Milman and Drapeau, 2012). Locke and Brawley (2018) found that people who make many exercise-related cognitive errors (ECEs), such as thinking that people are making fun of you because you are not doing an exercise correctly, focused on the challenging parts of described exercise, and interpreted descriptions of exercise negatively. Thus, people who make many ECEs may interpret Fitspiration differently than people who are less prone to such errors. For example, they may believe the messages more, and may be inclined to view exercise unfavorably because ECEs represent a biased view of exercise-relevant information that distorts how individuals perceive exercise (Locke and Brawley, 2016, 2018).

The primary purposes of this research were to examine the relationships of implicit and explicit attitudes and after viewing Fitspiration to believability and if ECEs moderated explicit attitudes or believability, or the relationship between implicit attitudes and believability (study one). The second study tested Fitspiration media compared to control media on intention to exercise with implicit attitudes, explicit attitudes, and believability examined as possible mediators, and cognitive errors as a possible moderator. In two separate studies, self-identified men and women were shown gender-specific Fitspiration media. The first study was primarily descriptive whereas the second study compared Fitspiration to control media using an experimental design.

## 2. Materials and methods common to both studies

### 2.1. Measures

All surveys are available in the supplemental file. Inquisit software^1^ was used to present all study materials and collect the data. 1 Data Availability Statement.

The datasets analyzed for this study can be found in University of Alberta dataverse [doi:10.5683/SP3/OKQGSR].

#### 2.1.1. Exercise-related cognitive error questionnaire

Seven items from the validated ECEQ were used to measure ECEs (Locke and Brawley, 2016). The ECEQ is comprised vignettes that depict cognitive error responses to common exercise challenges such as worries about being tired after not having exercised for a long time. Participants rated the degree to which their reactions were similar to how they would think, on a nine-point Likert scale ranging from 1 (not at all like I would think) to 9 (exactly like I would think). A higher score indicates the propensity to make more ECEs. The ECEQ demonstrated strong internal consistency in the current study, with internal reliabilities for women and men across both studies ranging from *α* = 0.83–0.95.

#### 2.1.2. Implicit attitudes

A single-category implicit association task (SC-IAT; Karpinski and Steinman, 2006) had participants categorize words as quickly as possible by pressing keys on a computer keyboard. The three categories were “exercise,” “good,” and “bad.” The eight exercise words reflected activities typically shown in Fitspiration (running, gym, strength training, biking, stairs, sports, interval training, and yoga). Sixteen words in the good category reflected language commonly used in Fitspiration text (capable, strong, dedicated, fit, successful, winner, proud, willpower, disciplined, motivated, toned, sexy, determined, pleasant, and deserving, fun). Sixteen words in the bad category also reflected common Fitspiration text (incapable, weak, lazy, unfit, unsuccessful, loser, ashamed, jealous, undisciplined, unmotivated, flabby, humiliated, failure, painful, ugly, and unpleasant). In one block, the exercise and good words shared the same response button and the bad words the other response button. This was reversed in the other block. The words were presented twice each per block in a random order and the block order counterbalanced across participants. Participants were shown a red X if they made an error and the next trial did not appear until they corrected their response. D-scores were calculated as a measure of implicitly measured exercise evaluations, according to the improved scoring algorithm (Greenwald et al., 2003). A higher score indicates a more positive implicit attitude. Reliability was assessed using intra-class correlations (ICC) of response times for correct odd and even trials and reliability was strong ranging from 0.70 to 0.92 across both studies and for women and men.

#### 2.1.3. Explicit attitudes

This was measured with a thought listing task during which participants were asked to list up to five thoughts (open-ended) they had while viewing the Fitspiration (or control media in study two) they just saw. This is a technique pioneered by Cacioppo and Petty (1981) and has since been used in advertising research. Responses were coded according to the *a priori* codes: pro-Fitspiration and anti-Fitspiration. Explicit attitudes toward Fitspiration were operationalized into a group variable: positive (only pro-Fitspiration comments were made), negative (only anti-Fitspiration comments were made), or mixed (both pro- and anti-Fitspiration comments).

#### 2.1.4. Believability

A 9-item scale asked participants to “Please judge the pictures you saw according to how YOU perceived them” on items including believable/unbelievable, trustworthy/untrustworthy, convincing/not convincing, and honest/dishonest (Beltramini and Evans, 1985). A mean of all items was used; items were scored so that a higher score indicates finding the Fitspiration or control media more believable. The internal reliability across both studies for the men’s and women’s data ranged from 0.93 to 0.95.

#### 2.1.5. Prior exposure to fitspiration or control media

This was assessed with the question: “Have you seen images like this before? (Yes/No). If yes, three additional questions were presented: (1) Do you actively search for these images (e.g., using #fitspiration on Instagram or Twitter)? (yes/no); (2) How frequently do you see images like these? (from ‘once a month or less’ to ‘daily’); and (3) Would you consider sharing images like this on social media sites such as Instagram or Twitter? (no, maybe, and yes).

#### 2.1.6. Demographics

Age, gender, education, ethnicity, and height and weight (used to calculate body mass index) were measured. Leisure-time physical activity (LTPA) in the first study was measured with one question assessing how often a participant engaged in moderate to vigorous physical activity for at least 30 min a day the last 3 months. Seven response options ranged from “not at all” to “four or more times a week.” In study two, LTPA was measured using the moderate and vigorous items from the Godin leisure-time exercise questionnaire (Godin, 2011). As advocated by Godin, a leisure-time moderate to vigorous physical activity (MVPA) score was calculated by multiplying the number of strenuous bouts by nine (approximate metabolic equivalent (MET) value) and the number of vigorous bouts by five (approximate MET value) and summing the two scores. A score < 14 is categorized as inactive, scores between 14 and 24 are moderately active, and scores >24 are considered active.

#### 2.1.7. Fitspiration and control media

The Fitspiration media used in both studies were found through searches using #Fitspiration and #Fitspo. Media with women and men, respectively, were found. Those used in this research were those that had images of people with in-lay text. The women shown were thin with toned bodies and bare midriffs. The in-lay text were sayings such as “Do not wish for it, work for it.” The men shown were very muscular, often with no shirts on and in-lay text were sayings such as “You do not find willpower, you create it.”

To determine the control media used in study two, two pilot tests were conducted, one with 30 messages that included images of women and one with 30 messages with images of men, drawn from the Fitspiration used in study one, fitness magazines, and general exercise advertising. All were images of people exercising in gyms or outdoors and showed a variety of body shapes. Each image had in-lay text with messages such as “the secret of getting ahead is getting started” or “create an active life for a healthy mind.” Participants rated each according to how much they agreed with the combined image and text message, rated on a scale of 1–7 and were also asked where they thought they would most likely see each image: in public health advertising, fitness magazines, or Fitspiration. Twenty-six women between the ages of 18 and 33 years (*M* = 24.38 [SD = 4.4]) rated the women’s messages and 26 men between the ages of 18 and 29 years (*M* = 23.15 [SD = 4.00]) rated the men’s messages. The final five Fitspiration and five control messages chosen for study two were selected based on representing different sources but were most similar in agreement ratings. This controlled for how appealing the media were to participants, and any differences in attitudes or believability were not based on personal appeal. There was quite a bit of overlap between Fitspiration and fitness magazine categorizations; therefore, the media selected for inclusion were those that a majority of participants categorized as most likely to be seen in a Fitspiration post. For the control condition, media were selected where there was no overlap in categorization and no significant differences in ratings of agreement with the message. This analysis is reported in more detail in the supplementary file, section 2.

#### 2.1.8. Data analysis

The thought listing responses were coded by one researcher then one-third of the responses were randomly selected using SPSS and coded by a second researcher to test intercoder-reliability. Kappa was calculated and any discrepancies were resolved through discussion. The final reliability ranged from 0.83 to 0.86.

Correlations between main study variables were conducted for descriptive purposes. Also for descriptive purposes differences and to determine whether activity level should be controlled for, differences between explicit attitudes (a categorical variable) and LTPA were tested using Chi-square analyses. Hayes (2018) conditional process analysis (using the PROCESS macro for SPSS; model 8) was used to test the moderated mediation models. Separate analyses were conducted for women and men in each study. Missing data on key variables were list-wise deleted for each analysis and independent and moderator variables were standardized prior to the analyses. Because multiple paths were tested, alpha was set to 0.01 for study one and 0.005 for study two. Significant relationships between explicit attitudes (a categorical variable) and continuous variables were followed up with Analysis of Variance tests (ANOVAs).

### 2.2. Study one

This study examined the moderating effect of ECEs on the believability of Fitspiration media. Implicit attitudes were the predictor and explicit attitudes the proposed mediator. The paths tested are shown in Figure 1. It was hypothesized that implicit attitudes would be positively related to explicit attitudes (H1) and believability (H2), that explicit attitudes would be positively related to believability (H3), and that ECEs would moderate these relationships such that participants who made more ECEs would show more positive explicit attitudes toward Fitspiration (H4), would find the media more believable (H5), and would have stronger relationships between implicit and explicit attitudes (H6), and between implicit attitudes and believability (H7).

#### 2.2.1. Methods

##### 2.2.1.1. Participants

Two hundred and ninety-two participants (151 women and 141 men) were recruited from a first-year psychology class participant pool. This research received ethics approval from a university research ethics board and all participants provided informed consent before participating. Separate studies were conducted for people who identify as men and women because fitspiration media are highly gendered. Therefore, different gender-specific media were shown to women and men. A sample size of 135 was calculated for each gender sample using G*Power 3.1.9.2 linear multiple regression (fixed model, *R*^2^ deviation from zero) test, with power (1 – *β* = 0.9), *a priori α* = 0.05, and medium effect size assumed.

##### 2.2.1.2. Procedures

All procedures were approved by a university research ethics board and all participants provided informed consent before participating. Participants were awarded one credit toward their final grade for participation and were given the option of completing an educational activity if they did not want to do the study (no participant chose this option). Participants were sent a debriefing document at the end of participation explaining the purpose of the study, including the possible negative effects of Fitspiration and why understanding their believability is important. Participants first completed the ECEQ and were then shown 20 Fitspiration images with in-lay text for 7 s each, and asked to rate each one on the question “How much do you agree with this combined image and message?” on a scale of 1 (do not agree at all) to 7 (completely agree). This was followed by the SC-IAT (measuring implicit attitudes), the thought listing task (measuring explicit attitudes), and the believability, prior exposure to fitspiration images, and demographics survey. The image ratings were used as a manipulation check and the data were not further analyzed.

#### 2.2.2. Results

All participants provided image ratings but 12 women and 18 men did not make a Fitspiration-related comment during the thought listing task and were excluded from the analyses, leaving final sample sizes of 139 women and 125 men. Demographic information is shown in Table 1. The majority of women (84.9%; *n* = 118) and men (76.4%; *n* = 94) reported having seen Fitspiration before; however, of participants who reported having seen it previously, 28.1% of women and 26.8% of men reporting seeing it once a month or less, and only 10 women and two men reported actively searching for it.

**Table 1:** Demographic information.

Demographic variable		Study one		Study two	
		Women (*N* = 139)	Men (*N* = 123)	Women (*N* = 195)	Men (*N* = 173)
Age in years M (SD; range)		18.83 (1.77; 18–33)	19.05 (1.89; 18–28); 1 missing	22.55 (3.43; 18–30)	24.16 (3.81; 18–33)
BMI M (SD)		24.68 (8.53); 15 missing	24.72 (7.98); 7 missing	27.22 (10.79); 17 missing	26.66 (8.74); 16 missing
LTPA N (%)^a^	Inactive	57 (41.0%)	41 (33.3%)	57 (29.2%)	
	Moderately active	25 (17.9%)	15 (12.2%)	38 (19.5%)	26 (15.0%)
	Active	57 (41 0.0%)	66 (53.7%)	98 (50.3%)	111 (64.2%)
	Did not report	0	1 (0.8%)	2 missing	12 (6.9%)
Ethnicity *N* (%)	Caucasian/white	56 (40.3%)	38 (30.9%)	130 (66.7%)	106 (61.3%)
	Asian	47 (33.8%)	57 (46.3%)	30 (15.4%)	27 (15.6%)
	Black	5 (3.6%)	8 (6.5%)	4 (2.1%)	17 (9.8%)
	Other/mixed	26 (18.7%)	16 (13.0%)	30 (15.3%)	12 (6.9%)
	Did not report	5 (3.6%)	4 (3.3%)	1 (0.5)	1 (0.6%)
Education *N* (%)	High school or less	All undergraduate students		59 (30.3%)	40 (23.1%)
	College or vocational			46 (23.6%)	37 (21.4%)
	Undergraduate			66 (33.8%)	61 (35.3%)
	Graduate or professional			21 (10.8%)	35 (20.2%)
	Missing or undisclosed			3 (1.5%)	0

a Study one, inactive were participants who reported being active only a few times a month or less, moderately active participants exercised 2–3 times a week and active participants were those who reported exercising more than three times a week; Study two categories are based on MET values as advised by Godin (2011).

##### 2.2.2.1. Women’s results

Correlations between continuous variables (i.e., implicit attitudes, ECEs, and believability) are shown in Table 2. Only believability was significantly negatively correlated with ECEs, *p* < 0.05. Twenty-six women made pro-Fitspiration comments only (“The pictures were very motivational”), 66 made anti-Fitspiration comments only (e.g., “They were very cheezy. A lot of them related your body appearance to how you should feel, and I did not like that”), and 47 made both positive and negative comments (e.g., “I’m used to seeing these kinds of pictures while looking for exercise routines on Google Images or Pinterest. I find some of them motivating, but others are just kind of silly or do not motivate me at all.”). There was no relationship between type of comment and LTPA, *X*^2^ = 0.63.

**Table 2:** Correlations between continuous variables in study one; Results for men are below the diagonal and the women’s results are above the diagonal.

	Implicit attitudes	Believability	Exercise-related cognitive errors
Implicit attitudes	-	0.060	−0.022
Believability	−0.057	-	−0.179*
Exercise-related cognitive errors	−0.133	−0.120	-

*p < 0.05.

The results of the moderated mediation are shown in Table 3. The overall models were not supported; the index of moderated mediation was not significant = 0.04 (95% CI = −0.08; 0.16). There was one significant relationship: between explicit attitudes and believability. The relationship between explicit attitudes and believability was explored with an ANOVA, *F* (2, 136) = 34.30, *p* < 0.001. Participants who made only pro-Fitspiration comments had significantly higher believability scores (*M* = 4.63 [SD = 0.98]) compared to those who made only anti-Fitspiration comments (*p* < 0.001; *M* = 2.64 [SD = 0.98]) with a large effect size, Cohen’s *d =* 2.03. Those who made mixed comments had significantly lower believability scores (*M* = 3.43 [SD = 1.17]) than those who made only pro-Fitspiration comments, *p* < 0.001, Cohen’s *d =* 1.11, and significantly higher believability scores compared to those who made only anti-Fitspiration comments, *p* < 0.001, Cohen’s *d =* 0.73.

**Table 3:** Study one results for women and men’s models.

Path	Women’s model				Men’s model			
	*B*	SE_b_	*t*	*p*	*B*	SE_b_	*t*	*p*
H1. Implicit attitudes to explicit attitudes	0.20	0.06	0.91	0.37	−0.36	0.25	−1.42	0.16
H2. Implicit attitudes to believability	0.37	0.38	0.97	0.33	−0.56	0.38	−1.46	0.15
H3. Explicit attitudes to believability	−0.41	0.15	−2.76	0.006	−0.57	0.14	−4.14	<0.001
H4. ECEs moderating explicit attitudes	0.01	0.03	0.15	0.88	−0.02	0.05	−0.37	0.71
H5. ECEs moderating believability	−0.12	0.06	−2.11	0.04	−0.13	0.08	0.76	0.09
H6. Implicit attitudes to explicit attitudes moderated by ECEs	−0.10	0.12	−0.82	0.41	0.09	0.17	0.52	0.60
H7. Implicit attitudes to believability moderated by ECEs	<0.01	0.20	−0.01	0.99	0.19	0.26	0.76	0.45

##### 2.2.2.2. Men’s results

Correlations between ECEs, implicit attitudes, explicit attitudes, and believability are shown in Table 2. There were no significant correlations. Fifty-one men made only pro-Fitspiration comments (e.g., “I thought they were cool. They are pushing me to work out more often and not give up”), 34 made only anti-Fitspiration comments (e.g., “privileged, not everybody is limitless -the idea that you have to be in extreme pain to make gains is stupid -success is a mindset though -these faces are off putting, calm down dude”), and 38 made both positive and negative comments (e.g., “Pictures about excuses had a good message. I disagreed with the pictures about nothing being able to stop you”). There was no relationship between type of comment and LTPA, *X*^2^ = 0.34.

The overall moderated mediation model was not supported and the index of moderated mediation was not significant = −0.05 (95% CI = −0.27; 0.14). The only significant relationship was between explicit attitudes and believability. This relationship was explored with an ANOVA, *F* (2, 120) = 44.73, *p* < 0.001. Participants who made only pro-Fitspiration comments had significantly higher believability scores (*M* = 4.98 [SD = 1.11]) compared to those who made only anti-Fitspiration comments (*p* < 0.001; *M* = 2.82 [SD = 1.01]), with a large effect size, Cohen’s *d =* 2.04. Those who made mixed comments had significantly lower believability scores (*M* = 4.04 [SD = 0.93]) than those who made pro-Fitspiration comments *p* < 0.001, Cohen’s *d =* 0.91, and significantly higher believability scores compared to those who made only anti-Fitspiration comments, *p* < 0.001, Cohen’s *d =* 1.25.

#### 2.2.3. Discussion

Most of the hypotheses were not supported, indicating no relationship between implicit attitudes toward the media and explicit attitudes or believability. ECEs did not moderate these relationships. The only hypothesis consistently supported was that explicit evaluations were positively related to believability. The thoughts reflected positive or negative attitudes that were related to motivation and went beyond what is measured by the believability scale. For example, one man commented that “They could help in boosting motivation.” This study was limited by an undergraduate student sample and did not include comparisons to other exercise-related media. Further, given that this is the first study to examine implicit attitudes toward Fitspiration, or to examine ECEs and believability, the study should be replicated before any conclusions can be reached. Therefore, study two replicated this study with a more diverse sample and further expanded to compare Fitspiration to other forms of exercise-related media and the effects on intention to exercise.

### 2.3. Study two

This research was designed to expand on study one by using an experimental design, a nonstudent sample, and to examine intention as the primary outcome. Also, questions were developed that specifically addressed cognitive errors that reflect the more extreme nature of Fitspiration. Thus, the moderating effects of both ECEs and Fitspiration-related cognitive errors (FCEs) were examined. A final difference was fewer media were used as it was found in Study one that there was little variability in agreement with the media and so participants viewed five images to reduce participant fatigue.

The primary purpose was to examine whether ECEs or FCEs moderated the effects of media type (Fitspiration compared to control) on implicit attitudes, explicit attitudes, or believability and if these mediated the effects of the media on intention to exercise. The model tested is shown in Figure 2. Separate models were tested with ECEs and FCEs as the potential moderator and again, separate studies were conducted for women and men. This study was approved by a university research ethics board and all participants provided informed consent before participating.

It was hypothesized that explicit attitudes (H1), implicit attitudes (H2), and believability (H3) would be higher for Fitspiration compared to control media; that ECEs and FCEs would moderate most of these relationships such that participants who made more ECEs or FCEs would show more positive explicit attitudes (H4), more positive implicit attitudes (H5), and find the media more believable (H6); that participants who made more ECEs or FCEs and were in the Fitspiration condition would have higher explicit attitudes (H7), implicit attitudes (H8), and believability (H9). Relative to intention to exercise, it was hypothesized that participants in the control condition would have greater intention (H10), and that higher intention would be found in participants with more positive explicit attitudes toward the media (H11), implicit attitudes toward the media (H12), or found the media more believable (H13); that participants who made more ECEs or FCEs would have lower intention (H14) and that the relationship of media type to intention would be moderated by ECEs or FCEs such that participants in the Fitspiration condition who made more cognitive errors would have greater intention to exercise compared to participants who made fewer cognitive errors or participants in the control condition (H15).

#### 2.3.1. Methods

##### 2.3.1.1. Participants

Women and men, aged 18–30 years, from the United Kingdom, the United States, Canada, New Zealand, and Australia, aged 18–65 years, were recruited using Prolific.^2^ Prolific is a research-focused platform that adheres to transparency using guidelines based on research ethics. Participants who completed the study received £5. To power our multiple regression analysis with the greatest number of independent variables, a sample size of 179 participants was needed for each gender. This was calculated using G*Power 3.1.9.2 the linear multiple regression (Fixed model, *R*^2^ deviation from zero test), with power (1 – *β* = 0.9), *a priori* α = 0.01 because of separate models for ECEs and FCEs, and a moderate effect size for the overall model. Further, because we ran two models for each gender study (one with ECEs as the moderator and the other with FCEs as the moderator), we set alpha to 0.025.

##### 2.3.1.2. Design and procedure

Participants were randomly assigned to view the Fitspiration or the control messages. Participants completed the ECEs and FCEs questionnaire at pretest. This was followed by viewing the Fitspiration or control media, immediately after which posttest measures were completed in the following order: the SC-IAT, thought listing, believability, exercise intention, prior exposure to similar messages, and demographics.

##### 2.3.1.3. Materials and measures

In addition to the materials reported earlier, the following measures were used for this study.

###### 2.3.1.3.1. Fitspiration-related cognitive errors

Seven items were created representing FCEs for the purposes of this study. All items were rated from 1 (not at all how I would think) through 5 (about half the time I would think like this) to 9 (almost exactly how I would think). A factor analysis that included the ECE and FCE items showed the ECE and FCE items differentiated with good internal reliability for both constructs for men and women (range = 0.81–0.88; see supplementary file, section 3, for complete results). Given these results, mean scores were created representing ECEs and FCEs.

###### 2.3.1.3.2. Intention

This was measured with one item: “I intend to exercise for at least 150 min a week,” rated as yes or no.

#### 2.3.2. Results

Demographic information for men and women are shown in Table 1. The majority of women (77.4%; *n* = 151) and men (69.9%; *n* = 121) reported having seen similar media before; however, of participants who reported having seen it previously, 20.5% of women and 27.2% of men reporting seeing it once a month or less, and only twenty women and 11 men reported actively searching for it.

##### 2.3.2.1. Women’s results

Two hundred and ninety-six women completed the study. Of these, 13 had missing survey data, and six did not provide any comments about the messages. An additional 82 made comments but they did not fall under the pro-fitspiration or anti-fitspiration codes (rather they were more general about exercise, presentation of the media, etc.…). After excluding the data from these participants, the data from 195 women were included in the analyses, 90 in the Fitspiration condition and 105 in the control condition. Consistent with the pilot test, there were no differences in how much the Fitspiration media were agreed with (M = 5.19 [SD = 1.01]) compared to the control media (M = 5.24 [SD = 1.03]), *F* (1, 275) = 0.205, *p* = 0.65. Among women who saw Fitspiration, 39 made only positive comments, 29 made only negative comments, and 22 made both positive and negative comments. In the control condition, 39 participants made only positive comments, 36 made only negative comments, and 30 made both positive and negative comments.

Correlations between continuous variables are shown in Table 4. Explicit attitudes were significantly negatively correlated with believability, *p* < 0.001, and FCEs *p* < 0.01, and FCEs were positively correlated with believability, *p* < 0.01. For descriptive purposes, simple t-tests were conducted with image group (Fitspiration or control) as the independent variable and ECEs, FCEs, implicit evaluations, and believability as the dependent variables. Chi-square analyses were used to examine the differences in explicit attitudes by image group. Significance was set to *p* < 0.01 because of multiple tests. As in study one, explicit attitudes were negatively related to believability.

**Table 4:** Correlations between continuous variables in study two; Results for men are below the diagonal and the women’s results are above the diagonal.

	Implicit attitudes	Explicit attitudes	Believability	ECEs	FCEs	Difference by media type
Implicit attitudes	-	−0.038	−0.047	−0.131	0.019	*p* = 0.70
Explicit attitudes	0.153*	-	−0.285***	−0.041	−0.156*	*p* = 0.66
Believability	0.046	−0.440***	-	−0.100	0.165*	*p* = 0.66
ECEs	−0.219**	0.082	−0.187*	-	−0.053	*p* = 0.05
FCEs	−0.004	−0.159*	0.288**	0.017	-	*p* = 0.64
Difference by media type	*p* = 0.04	*p* = 0.06	*p* = 0.43	*p* = 0.44	*p* = 0.91	-

*p < 0.05; **p < 0.01; ***p < 0.001.

ECEs, Exercise-related cognitive errors; FCEs, Fitspiration-related cognitive errors.

The results of the moderated mediation model with ECEs as the moderator are shown in Table 5 and with FCEs as the moderator are shown in Table 6. Neither overall model was supported. None of the indices of moderated mediation were significant. In the models with ECEs as the moderator:, the index of moderated mediation for media type on intention mediated by explicit attitudes in the women’s model <0.001 (95% CI = −0.05; 0.06). The index of moderated mediation for media type on intention mediated by implicit attitudes <0.001 (95% CI = −0.05; 0.05). The index of moderated mediation for media type on intention mediated believability =0.04 (95% CI = −0.03; 0.14). In the models with FCEs as the moderator, the index of moderated mediation for media type on intention mediated by explicit attitudes <0.001 (95% CI = −0.0; 0.0). The index of moderated mediation for media type on intention mediated by implicit attitudes <0.001 (95% CI = −0.05; 0.04). The index of moderated mediation for media type on intention mediated believability = −0.02 (95% CI = −0.13; 0.06).

**Table 5:** Study two results with ECEs as moderator; women’s and men’s models.

Path	Women’s model				Men’s model			
	*B*	SE_b_	*t*	*p*	*B*	SE_b_	*t*	*p*
H1. Media type to explicit attitudes	−0.11	0.12	−0.91	0.36	0.19	0.12	1.60	0.11
H2. Media type to implicit attitudes	−0.01	0.04	−0.17	0.87	0.10	0.05	1.88	0.06
H3. Media type to believability	−0.05	0.09	−0.24	0.81	−0.20	0.21	−0.97	0.34
H4. ECEs moderating explicit attitudes	0.09	0.11	0.85	0.40	0.03	0.10	0.32	0.75
H5. ECEs moderating implicit attitudes	−0.02	0.04	−0.59	0.56	−0.12	0.04	−2.76	0.006
H6. ECEs moderating believability	−0.23	1.07	−1.62	0.11	−0.51	0.18	−2.85	<0.005
H7. Media type to explicit attitudes moderated by ECEs	−0.07	0.07	−0.99	0.32	0.004	0.06	0.06	0.95
H8. Media type to implicit attitudes moderated by ECEs	<0.01	0.03	0.02	0.98	0.06	0.03	2.03	0.04
H9. Media type to believability moderated by ECEs	0.14	0.12	1.22	0.22	0.24	0.11	2.18	0.03
Path	*B*	SE_b_	*Z*	*p*	*B*	SE_b_	*Z*	*p*
H10. Media type to intention	0.30	0.42	0.71	0.48	−0.55	0.52	−1.05	0.29
H11. Explicit attitudes to intention	−0.05	0.24	−0.19	0.85	−0.24	0.31	−0.77	0.44
H12. Implicit attitudes to intention	−0.33	0.62	−0.53	0.60	0.90	0.65	1.38	0.17
H13. Believability to intention	−0.31	0.15	2.06	0.04	0.001	0.18	0.01	0.995
H14. ECEs moderating intention	−0.53	0.41	−1.29	0.20	−1.03	0.46	−2.21	0.03
H15. Media type to intention moderated by ECEs	−0.08	0.28	−0.28	0.78	0.35	0.27	1.29	0.20

**Table 6:** Study two results with FCEs as moderator; women’s and men’s models.

	Women’s model				Men’s model			
Path	*B*	SE_b_	*t*	*p*	*B*	SE_b_	*t*	*p*
H1. Media type to explicit attitudes	−0.11	0.12	−0.95	0.34	0.19	0.12	1.58	0.12
H2. Media type to implicit attitudes	−0.02	0.04	−0.43	0.67	0.10	0.05	1.95	0.05
H3. Media type to believability	−0.09	0.19	−0.47	0.64	−0.18	0.21	−0.89	0.37
H4. FCEs moderating explicit attitudes	−0.07	0.11	−0.62	0.54	−0.10	0.13	−0.73	0.46
H5. FCEs moderating implicit attitudes	0.04	0.04	0.98	0.33	−0.07	0.06	−1.23	0.22
H6. FCEs moderating believability	0.25	0.18	1.36	0.18	0.48	0.24	2.06	0.04
H7. Media type to explicit attitudes moderated by FCEs	−0.01	0.07	−0.10	0.92	.0.01	0.08	0.17	0.87
H8. Media type to implicit attitudes moderated by FCEs	−0.03	0.03	−0.96	0.34	0.04	0.04	1.17	0.24
H9. Media type to believability moderated by FCEs	−0.07	0.11	−0.65	0.51	−0.14	0.15	−0.95	. 43
Path	*B*	SE_b_	*Z*	*p*	*B*	SE_b_	*Z*	*p*
H10. Media type to intention	0.08	0.35	0.70	0.48	−0.21	0.42	−0.50	0.62
H11. Explicit attitudes to intention	0.15	0.23	0.67	0.05	−0.25	0.29	−0.87	0.39
H12. Implicit attitudes to intention	0.14	0.57	0.25	0.80	1.38	0.60	2.31	0.02
H13. Believability to intention	0.34	0.14	2.40	0.02	0.08	0.17	0.47	0.64
H14. FCEs moderating intention	0.01	0.33	0.05	0.96	−1.03	0.46	−2.21	0.03
H15. Media type to intention moderated by FCEs	0.17	0.21	0.82	0.41	0.40	0.29	−1.41	0.16

There were no significant relationships in either model (i.e., with ECEs or FCEs as the moderator).

##### 2.3.2.2. Men’s results

Three hundred and ten men completed the study. Of these, thirty had missing survey data, and eight did not provide any comments about the messages. An additional 92 made comments, but they did not fall under the pro-fitspiration or anti-fitspiration codes. After excluding the data from these participants, the data from 173 men were included in the analyses, 90 in the fitspiration condition and 83 in the control condition. There were no differences in how much the Fitspiration media were agreed with (M = 4.78 [SD = 1.32]) compared to the control media (M = 4.93 [SD = 1.72]), *F* (1, 270) = 0.63, *p* = 0.43. Among men who saw Fitspiration, 45 made only positive comments, 23 made only negative comments, and 22 made both positive and negative comments. Among men in the control condition, 46 made only positive comments, 27 made only negative comments, and 9 made both positive and negative comments.

Correlations between continuous variables are shown in Table 4. ECEs were negatively correlated with implicit attitudes, *p* < 0.001, and believability, *p* < 0.01, and believability was also negatively correlated with explicit attitudes, *p* < 0.001, and positively correlated with FCEs, *p* < 0.01.

The results of the moderated mediation model with ECEs as the moderator are shown in Table 5 and with FCEs as the moderator are shown in Table 6. Neither overall model was supported. None of the indices of moderated mediation were significant. In the models with ECEs as the moderator, the index of moderated mediation for media type on intention mediated by explicit attitudes <0.001 (95% CI = −0.05; 0.06). The index of moderated mediation for media type on intention mediated by implicit attitudes <0.001 (95% CI = −0.02; 0.17). The index of moderated mediation for media type on intention mediated believability <0.001 (95% CI = −0.12; 0.11). In the models with FCEs as the moderator, the index of moderated mediation for media type on intention mediated by explicit attitudes <0.001 (95% CI = −0.07; 0.06). The index of moderated mediation for media type on intention mediated by implicit attitudes = 0.06 (95% CI = −0.0; 0.22). The index of moderated mediation for media type on intention mediated = 0.01 (95% CI = −0.10; 0.05).

In the model with ECEs as the moderator, ECEs moderated believability. Higher ECEs were negatively related to implicit attitudes and believability (see Table 5).

In the model with FCEs as the moderator, there were no significant relationships.

#### 2.3.3. Discussion

This study examined more possible mechanisms through which exercise-related media may affect people’s exercise intention. There were no significant relationships in the women’s study with the exception that explicit attitudes were negatively related to believability. Previous researchers found that a factually incorrect blog post about how women’s appearance can change with exercise was rated as more believable than a factually correct blog post (Ori et al., 2021). However, the current study found no difference in believability between the Fitspiration compared to control media even though the control messages were largely about exercising for positive mental health and happiness, whereas the Fitspiration messages implied that the featured bodies could be achieved through exercise. It may be that both messages are equally palatable to women.

In the men’s models, higher ECEs were negatively related to believability regardless of which condition the men were in. This is a consistent finding in this research across both studies (albeit with small correlations) and is opposite to the hypothesized relationship. Locke and Brawley (2016) found that participants who made more ECEs focused on the vignette aspects that would hinder exercise. Thus, it is possible that ECEs were negatively related to Fitspiration believability because the text included was about success and effort, which may have seemed unattainable for someone predisposed to worrying about being tired or being made fun of when exercising. FCEs were not related to believability among women or men.

There were no relationships between ECEs or FCEs and implicit associations which corroborates other research finding no relationship between ECEs and automatic affective evaluations of exercise (Locke and Berry, 2021). Further, none of the constructs tested were related to intention. Fitspiration targeting men and women, including the media used in the current research, tends to feature idealized bodies and highlight appearance as the reason to exercise (Boepple et al., 2016). Researchers have found that viewing such media was related to greater inspiration to exercise (Tiggemann and Zaccardo, 2015), but the current research did not find a relationship between Fitspiration and exercise intention. Other researchers also report that Fitspiration media featuring an athletic ideal may have influenced exercise motivation, but the motivation did not translate to short-term exercise behavior (measured as distance covered during a 10-min treadmill test; Robinson et al., 2017). Thus, this research again shows that it is unlikely that “fitspiration” inspires many people to exercise.

## 3. General discussion

This research explored if believability of Fitspiration messages was mediated by implicit attitudes toward exercise or explicit attitudes toward Fitspiration and if ECEs moderated the relationships (study one). Study two examined the relationship of implicit and explicit attitudes and believability of Fitspiration compared to control messages on exercise intention. ECEs were again tested as a possible moderator, but FCEs were also tested as a possible moderator. Believability was a key construct examined in this research because it relates to how convincing or truthful the media are thought to be. Therefore, this research was designed with the supposition that believability of Fitspiration is an important factor to understand because of the extreme bodies and messages that it presents. If someone finds Fitspiration convincing, it is a possible modifiable path for mitigating the negative effects found by a number of other researchers (e.g., Fatt et al., 2019; Griffiths and Stefanovski, 2019; Yee et al., 2020; Fioravanti et al., 2021). The majority of hypothesized paths were not supported.

Others have found that men who view more Fitspiration images had higher tendencies to internalize muscular ideals and to compare their appearance to others, which was related to less motivation to exercise for health (Fatt et al., 2019). Fatt et al. (2019) highlight that the majority of research has examined the effects of Fitspiration media on women, with less research with men. In the current research, proportionally more men than women had only positive explicit attitudes toward the Fitspiration media (42.6% of men in study one and 50% of men in the Fitspiration condition in study two compared to 18.7% women in study one and 43.3% in the Fitspiration condition in study two). It may be that because women have received more interventions intended to mitigate the effects of such media through body-positive movements (Scharrer, 2013), fewer women than men positively evaluated the media. The Fitspiration media showed bodies that conform to extreme ideas of what women and men ideally look like in Western culture. The exclusion of men from many body-positive interventions thus may result in more men wishing to conform to societal expectations of strong and muscular men (Eagly and Wood, 2013). This indicates that, as Fatt et al. proposed, the effects of Fitspiration on men should not be ignored and more interventions specific to men should be developed. However, the relationship of Fitspiration media to short and long-term exercise intention and behavior remains to be determined. There are likely other moderators to investigate that lead to either obsessive exercise or a lack of intention to exercise at all.

Another consistent finding was that explicit attitudes, represented by comments made about the messages, were related to believability. Participants who made only positive comments about the media reported them to be more believable, compared to those who made only negative comments, or a combination of positive and negative comments. In addition to providing further validation of the believability construct, this finding may indicate that the Fitspiration media may result in being motivated to achieve a certain appearance standard if one finds the messages believable. Therefore, positively endorsing and believing fitspiration may lead to negative outcomes including poorer body image (Tiggemann and Zaccardo, 2015; Fatt et al., 2019). Some participants found the messages off-putting. For example, a woman who made an anti-Fitspiration comment wrote, “I felt like the pictures were very cliche sayings and did not promote exercise in a healthy way.” It may be that for these women, Fitspiration reinforces unattainable and exaggerated exercise outcomes but this was not related to exercise intention. The effects of such media on intention to be active may be limited.

There were no relationships between implicit attitudes toward exercise and believability. Other research has shown a positive relationship between implicit attitudes and exercise behavior only among participants with low inhibitory control; that is those who are less able to suppress automatic thoughts or impulses (Padin et al., 2017). Thus, future research should investigate if increasing inhibitory control or other self-regulatory processes moderate critical evaluation of exercise messages. The current research also found no relationship between implicit attitudes toward exercise and explicit attitudes toward the media. Although the SC-IAT in the current research used words drawn from the Fitspiration text, these findings again raise the question of whether it is images or text (or a combination) that influence consumers. The words may have elicited associations not necessarily directly related to the images or text seen, whereas the explicit evaluations were directly related. Associations in memory may include a variety of representations (Deutsch et al., 2017). Thus, the media presented in the current research may have activated associations not measured, such as how exercise feels or specific exercise situations. This relates to general questions about the validity of IATs in general. Schimmack (2019) provided an overview of the lack of construct validity for the IAT and highlighted that what processes are actually measured remains debatable. Thus, given these concerns, the results of the current study require replication with multiple measures of associative processes before any firm conclusions can be reached.

Strengths of this research include two studies, one with a large sample with diverse educational background allowing for greater generalizability. This research thus informs possible mechanisms, including believability, of Fitspiration and other exercise media among younger adults in some Western nations. However, a few limitations should be noted. First, the SC-IAT only had words as stimuli and not images. If images of exercise were shown, there may have been different responses, and as previously noted, there are questions regarding the construct validity of such tests (Schimmack, 2019). Also, the research was conducted over the internet and there was no control over where the research was conducted. The context in which participants did the study (e.g., if others were in the same place, noise) may have influenced the results. Another limitation is that a dichotomous measure of intention was used, which may have reduced variability. Finally, we deliberately tried to control for how much the messages in the two conditions in study two were agreed with; however, it is possible that in doing so, the messages were too similar, thus limiting the ability to find differences in their effects on intentions to exercise. Future research should try to examine these aspects separately.

Overall, this study adds to the research on Fitspiration by identifying and excluding factors that predict its believability and the role that factors such as cognitive errors may play in that. It also demonstrated different results between men and women, highlighting that Fitspiration-related research should not assume similar mechanisms operate for women compared to men. Interventions to reduce the negative impacts of Fitspiration are needed for those who believe these messages.

## Data availability statement

The datasets presented in this study can be found in online repositories. The names of the repository/repositories and accession number(s) can be found at: https://borealisdata.ca/dataset.xhtml?persistentId=doi:10.5683/SP3/OKQGSR

## Ethics statement

The studies involving human participants were reviewed and approved by University of Alberta Research Ethics Board 2. Written informed consent for participation was not required for this study in accordance with the national legislation and the institutional requirements.

## Author contributions

TB designed the study, analyzed data, and wrote the first draft of the manuscript. SL and EO helped design the study, analyzed data, and contributed to manuscript writing. All authors contributed to the article and approved the submitted version.

## Funding

This research was funded by the Social Sciences and Humanities Research Council of Canada.

## Conflict of interest

The authors declare that the research was conducted in the absence of any commercial or financial relationships that could be construed as a potential conflict of interest.

## Publisher’s note

All claims expressed in this article are solely those of the authors and do not necessarily represent those of their affiliated organizations, or those of the publisher, the editors and the reviewers. Any product that may be evaluated in this article, or claim that may be made by its manufacturer, is not guaranteed or endorsed by the publisher.

## Footnotes

^1^ www.millisecond.com

^2^ https://www.prolific.co/about

## References

[ref1] Beltramini R. F. Evans K. R. (1985). Perceived believability of research results information in advertising. J. Advert. 14, 18–31. doi: 10.1080/00913367.1985.10672953

[ref2] Berry T. R. Jones K. E. McLeod N. C. Spence J. C. (2011). The relationship between implicit and explicit believability of exercise-related messages and intentions. Health Psychol. 30, 746–752. doi: 10.1037/a0025082, PMID: 21843000

[ref3] Boepple L. Ata R. N. Rum R. Thompson J. K. (2016). Strong is the new skinny: a content analysis of fitspiration websites. Body Image 17, 132–135. doi: 10.1016/j.bodyim.2016.03.001, PMID: 27045871

[ref4] Cacioppo J. T. Petty R. E. (1981). “Social psychological procedures for cognitive response assessment: the thought listing technique” in Cognitive Assessment. eds. Merluzzi T. Glass C. Genest M. (New York: Guilford), 309–342.

[ref5] De Houwer J. Gawronski B. Barnes-Holmes D. (2013). A functional-cognitive framework for attitude research. Eur. Rev. Soc. Psychol. 24, 252–287. doi: 10.1080/10463283.2014.892320

[ref6] Deutsch R. Gawronski B. Hofmann W. (2017). “Reflection and impulse: a framework for basic research and applied science” in Reflective and Impulsive Determinants of Human Behavior. eds. Deutsch R. Gawronski B. Hofmann W. (New York: Routledge), 51–68.

[ref7] Eagly A. H. Wood W. (2013). The nature–nurture debates: 25 years of challenges in understanding the psychology of gender. Perspect. Psychol. Sci. 8, 340–357. doi: 10.1177/1745691613484767 26172976

[ref8] Fatt S. J. Fardouly J. Rapee R. M. (2019). #malefitspo: links between viewing fitspiration posts, muscular-ideal comparisons, body satisfaction, and exercise motivation in men. New Media Soc. 21, 1311–1325. doi: 10.1177/1461444818821064

[ref9] Fioravanti G. Tonioni C. Casale S. (2021). #fitspiration on Instagram: the effects of fitness-related images on women’s self-perceived sexual attractiveness. Scand. J. Psychol. 62, 746–751. doi: 10.1111/sjop.12752, PMID: 34170526 PMC8518738

[ref10] Gawronski B. De Houwer J. (2014). “Implicit measures in social and personality psychology” in Handbook of Research Methods in Social and Personality Psychology. eds. Reis H. T. Judd C. M.. 2nd ed (New York: Cambridge University Press), 283–310.

[ref11] Godin G. (2011). The Godin-Shephard leisure-time physical activity questionnaire. Health Fitness J. Canada 4, 18–22.

[ref12] Greenwald A. G. Nosek B. A. Banaji M. R. (2003). Understanding and using the implicit association test: I. an improved scoring algorithm. J. Pers. Soc. Psychol. 85, 197–216. doi: 10.1037/0022-3514.85.2.197, PMID: 12916565

[ref13] Griffiths S. Stefanovski A. (2019). Thinspiration and fitspiration in everyday life: an experience sampling study. Body Image 30, 135–144. doi: 10.1016/j.bodyim.2019.07.002, PMID: 31299608

[ref14] Hayes A. F. (2018). Introduction to Mediation, Moderation, and Conditional Process Analysis: A Regression Based Approach. New York: Guilford Press.

[ref15] He J. Sun S. Zickgraf H. F. Lin Z. Fan X. (2020). Meta-analysis of gender differences in body appreciation. Body Image 33, 90–100. doi: 10.1016/j.bodyim.2020.02.011, PMID: 32151993

[ref16] Karpinski A. Steinman R. B. (2006). The single category implicit association test as a measure of implicit social cognition. J. Pers. Soc. Psychol. 91, 16–32. doi: 10.1037/0022-3514.91.1.16, PMID: 16834477

[ref17] Locke S. R. Berry T. R. (2021). Examining the relationship between exercise-related cognitive errors, exercise schema, and implicit associations. J. Sport Exerc. Psychol. 43, 345–352. doi: 10.1123/jsep.2021-0031, PMID: 34157673

[ref18] Locke S. R. Brawley L. R. (2016). Development and initial validity of the exercise-related cognitive errors questionnaire. Psychol. Sport Exerc. 23, 82–89. doi: 10.1016/j.psychsport.2015.11.003

[ref19] Locke S. R. Brawley L. R. (2018). Making one-sided exercise decisions: the influence of exercise-related cognitive errors. J. Health Psychol. 23, 1240–1249. doi: 10.1177/1359105316648485, PMID: 27270683

[ref20] Maibach E. (2007). The influence of the media environment on physical activity: looking for the big picture. Am. J. Health Promot. 21, 353–62, iii. doi: 10.4278/0890-1171-21.4s.353, PMID: 17465181

[ref21] Milman E. Drapeau M. (2012). Cognitive errors in cognitive behavioural therapy: a survey of researchers and practitioners and an assessment of the face validity of the cognitive error scale. J. Cogn. Behav. Psychother. 12:125e138.

[ref22] O’Cass A. Griffin D. (2006). Antecedents and consequences of social issue advertising believability. J. Nonprofit Public Sector Market. 15, 87–104. doi: 10.1300/J054v15n01_05

[ref23] Ori E. M. Berry T. R. Yun L. (2021). The believability of exercise blogs among young adults. J. Sport Exerc. Psychol. 43, 53–60. doi: 10.1123/jsep.2020-0177, PMID: 33412515

[ref24] Padin A. C. Emery C. F. Vasey M. Kiecolt-Glaser J. K. (2017). Self-regulation and implicit attitudes toward physical activity influence exercise behavior. J. Sport Exerc. Psychol. 39, 237–248. doi: 10.1123/jsep.2017-0056, PMID: 28937320 PMC6521970

[ref25] Pokhrel P. Herzog T. A. Fagan P. Unger J. B. Stacy A. W. (2019). E-cigarette advertising exposure, explicit and implicit harm perceptions, and e-cigarette use susceptibility among nonsmoking young adults. Nicotine Tob. Res. 21, 127–131. doi: 10.1093/ntr/nty030, PMID: 29444275 PMC6610163

[ref26] Prichard I. Kavanagh E. Mulgrew K. E. Lim M. S. C. Tiggeman M. (2020). The effect of Instagram #fitspiration images on young women’s mood, body image, and exercise behaviour. Body Image 33, 1–6. doi: 10.1016/j.bodyim.2020.02.00 32062021

[ref27] Robinson L. Prichard I. Nikolaidis A. Drummond C. Drummond M. Tiggeman M. (2017). Idealised media images: the effect of fitspiration on body satisfaction and exercise behaviour. Body Image 22, 65–71. doi: 10.1016/j.bodyimg.2017.06.001, PMID: 28654826

[ref28] Scharrer E. L. (2013). “Representations of gender in the media” in The Oxford Handbook of Media Psychology. ed. Dill K. E. (Oxford: Oxford University Press), 267–284.

[ref29] Schimmack U. (2019). The implicit association test: a method in search of a construct. Perspect. Psychol. Sci. 16, 396–414. doi: 10.1177/17456916198637 31647752

[ref30] Tiggemann M. Zaccardo M. (2015). “Exercise to be fit, not skinny”: the effect of fitspiration imagery on women’s body image. Body Image 15, 61–67. doi: 10.1016/j.bodyim.2015.06.003, PMID: 26176993

[ref31] Tiggemann M. Zaccardo M. (2018). ‘Strong is the new skinny’: a content analysis of #fitspiration images on Instagram. J. Health Psychol. 23, 1003–1011. doi: 10.1177/1359105316639436, PMID: 27611630

[ref32] Vandenbosch L. Fardouly J. Tiggemann M. (2022). Social media and body image: recent trends and future directions. Curr. Opin. Psychol. 45:101289. doi: 10.1016/j.copsyc.2021.12.002, PMID: 35030460

[ref33] Wood W. Eagly A. H. (2012). “Biosocial construction of sex differences and similarities in behavior” in Advances in Experimental Social Psychology. eds. Olson J. M. Zanna M. P., vol. 46 (London: Elsevier), 55–123.

[ref34] Yee Z. W. Griffiths S. Fuller-Tyszkiewicz M. Blakea K. Richardson B. Kruga I. (2020). The differential impact of viewing fitspiration and thinspiration images on men’s body image concerns: an experimental ecological momentary assessment study. Body Image 35, 96–107. doi: 10.1016/j.bodyim.2020.08.008, PMID: 32977202

