# Supplementary material for: Effects of fitspiration on believability and intention to exercise
Source: Front Psychol. 2023 Feb 20;14:1094276. doi: 10.3389/fpsyg.2023.1094276 (PMC9986607; doi:10.3389/fpsyg.2023.1094276)
Supplement: Supplementary file 1 [file Table_1.DOCX]

Supplementary Material

# Surveys

**Exercise-related cognitive errors**

**Instructions:**

The following short scenarios represent people’s reactions to different situations they might encounter when trying to exercise. Please indicate the degree to which the reactions in the following scenarios are similar to how you would think.

The following scenarios might not be exactly applicable to you and your situation

(Example: while the type of sport depicted in the scenario is one you would never consider doing, you could still react to the scenario).

Please try to put yourself in the situation and rate how similar the thought expressed in the scenario is to how you might react **using the scale below**. If the scenario is absolutely not applicable to you, please leave it blank.

| **1** | **2** | **3** | **4** | **5** | **6** | **7** | **8** | **9** |
| --- | --- | --- | --- | --- | --- | --- | --- | --- |
| **Not at all like I would think** |  |  |  | **About half of the time I would think like this** |  |  |  | **Almost exactly like I would think** |

_____You are considering starting to cycle with a local club. Every time you consider going to the club to join, you think to yourself, “I haven’t biked in years, I’m going to get way too tired to even be able to finish the ride.”

_____You are having a pretty busy week. You plan to exercise tonight, but when you get home from work you think to yourself, “I cannot justify exercising because I have so many other things to do.”

_____Because your exercise class is cancelled this week, you think to yourself, “I’m going to take the week off because I have no exercise class”.

_____You know the health the benefits of exercise, but think to yourself, “exercising is a big drain on all the other fun things I could be doing.”

_____You’ve been exercising for a few weeks. However, you’re getting frustrated because you aren’t seeing changes and the exercises aren’t getting easier. You think to yourself, “this is way too hard and no fun and decide to stop going to the gym.”

_____You have been feeling down and even depressed all day, you think to yourself, “I should just stay home instead of going to the gym today.”

_____You feel awkward and lost in the first gym/fitness class you attend. You think to yourself, “I feel so uncomfortable that I don’t want to go back.”

**Fitspiration-related cognitive errors (study 2 only)**

**Instructions:**

The following short scenarios represent people’s reactions to different situations they might encounter when trying to exercise. Please indicate the degree to which the reactions in the following scenarios are similar to how you would think.

The following scenarios might not be exactly applicable to you and your situation

(Example: while the type of sport depicted in the scenario is one you would never consider doing, you could still react to the scenario).

Please try to put yourself in the situation and rate how similar the thought expressed in the scenario is to how you might react **using the scale below**. If the scenario is absolutely not applicable to you, please leave it blank.

| **1** | **2** | **3** | **4** | **5** | **6** | **7** | **8** | **9** |
| --- | --- | --- | --- | --- | --- | --- | --- | --- |
| **Not at all like I would think** |  |  |  | **About half of the time I would think like this** |  |  |  | **Almost exactly like I would think** |

_____"When feeling too busy to workout you think to yourself, “don’t complain about things you’re not willing to work hard for.

_____To motivate yourself when you’re tired you think, “the only way to meet my fitness goals are through pain.”

_____To guide all of your decisions about what to eat and when to exercise you think, “my body is a reflection of my lifestyle”.

_____If you had a mediocre workout you think to yourself, “I’m never going to accomplish my fitness goals this way.”

_____When trying to motivate yourself after a long day a work you think to yourself, “if I don’t push myself to the max, I can’t make progress..”

_____When thinking about whether or not to go the gym you think, 'there is no success without sacrifice'.”

_____You get home and you don’t feel link going to the gym, to motivate yourself you think 'I have to stop making excuses if I want to or I will never see results.”

**Thought listing task**

Using the text box, please write up to five thoughts you had while viewing the pictures you looked at.

**Believability**

We need you to judge the images you just saw on a series of descriptive scales according to how YOU perceive the message given.

Make each item a separate and independent judgment. Do not worry or puzzle over individual items. It is your first impressions, the immediate feelings about the items that we want.

**The messages are:**

believable :__1__:__2__:__3__:__4__:__5__:__6__:__7__: not believable

trustworthy :__1__:__2__:__3__:__4__:__5__:__6__:__7__: not trustworthy

convincing :__1__:__2__:__3__:__4__:__5__:__6__:__7__: unconvincing

reasonable :__1__:__2__:__3__:__4__:__5__:__6__:__7__: unreasonable

honest :__1__:__2__:__3__:__4__:__5__:__6__:__7__: dishonest

unquestionable :__1__:__2__:__3__:__4__:__5__:__6__:__7__: questionable

conclusive :__1__:__2__:__3__:__4__:__5__:__6__:__7__: inconclusive

authentic :__1__:__2__:__3__:__4__:__5__:__6__:__7__: inauthentic

likely :__1__:__2__:__3__:__4__:__5__:__6__:__7__: unlikely

**Intentions (study 2 only).** “I intend to exercise for at least 150 minutes a week.” (Yes/No).

**Prior Exposure**

Have you seen images like this before? (Yes/No).

If yes

1) Do you actively search for these images (e.g., using #fitspiration on Instagram or Twitter)? (yes/no);

2) How frequently do you see images like these? (from ‘once a month or less’ to ‘daily’)

3) Would you consider sharing images like this on social media sites such as Instagram or Twitter? (no, maybe, yes).

**Demographics**

What is your age in years? ____________________

What is your height? _________ in feet and inches, or centimeters (circle one)

What is your weight? _________ in pounds or kilograms (circle one)

What is your ethnicity?____________________

Study 1: “Within the last 3 months, how often did you participate in one or more physical activities of moderate to vigorous intensity, totalling at least 30 minutes in a same day during your leisure time?” (7 response options from ‘not at all’ to ‘four or more times a week’; ).

Study 2: **Godin Leisure-Time Exercise Questionnaire**

- "The following questions relate to the amount of physical activity you do at different intensities. Please enter 0 if your answer is zero.
- Considering a typical 7-day period, how many times on average do you do the following kinds of exercise for more than 15 minutes in your free time?
- MODERATE physical activity (increase in heart rate, not exhausting - fast walking, baseball, easy bicycling, etc.)
- VIGOROUS physical activity (maximal effort, heart beats rapidly, sweating - e.g., running, hockey, soccer, etc.)

# Pilot study results

The women’s fitspiration showed thin, toned women with bare abdominal muscles. The text included sayings such as “excuses don’t burn calories” or “30-60 minutes of being uncomfortable is better than being uncomfortable your whole life.” The control messagesshowed women of diverse body sizes wearing tights and tops that covered their midriffs. The text included “be active for your mental health” or “your body deserves the best.” The men’s fitspiration showed men with very muscular bodies either shirtless or wearing a sleeveless shirt. The superimposed sayings included “I never dreamed about success, I worked for it!” and “sweat + sacrifice = success”. The control messages in the men’s study included men of diverse body sizes wearing shorts and either t-shirts or sweatshirts. The sayings included “a healthy life is a healthy mind” and “exercise improves your mood and boosts your confidence”.

To check there were no differences in how much the messages were agreed with, Repeated Measures ANOVAs were conducted with the mean rating for each type of image as the within subject’s factor. There was no difference in the ratings of the fitspiration messages (M = 4.50 [SD = 1.24]) compared to control messages (M = 4.58 [SD = 1.29]) for women, F (1, 25) = .07, *p* = .80, or in the ratings of the fitspiration (M = 3.95 [SD = 1.63]) compared to control messages (M = 4.08 [SD = 1.13]) for men, F (1, 25) = .20, *p* = .66.

# Factor analysis of ECE and FCE items.

A principal components analysis with the ECE and FCE items was conducted to determine factor validity. The results for the men’s data showed two factors with eigen values greater than 1, = 4.2 and 3.51, accounting for 54.7% of the variance. Varimax rotation showed ECE items all loaded on one factor with factor loadings > .66 and FCE items loaded on the second factor with factor loadings > .62. Reliability was good with $\alpha$ for ECEs = .83 and for FCEs $\alpha$= .85. The results for the women’s data showed two factors with eigen values = 4.2 and 3.35 accounting for 51.2% of the variance. There was one other factor with eigenvalue > 1 (1.06 accounting for 7.1% of the variance). A varimax rotation restricted to two factors showed ECE items all loaded on one factor with factor loadings > .38 and FCE items loaded on the second factor with factor loadings > .65. Reliability was good with $\alpha$ for ECEs = .88 and for FCE $\alpha$= .85.
